# Supplementary material for: Dynamics of local B cell migration during affinity maturation in the human tonsil
Source: bioRxiv. 2025 Nov 3:2025.10.31.685876. Preprint. [Version 1] doi: 10.1101/2025.10.31.685876 (PMC12637461; doi:10.1101/2025.10.31.685876)
Supplement: Supplement 1 [file NIHPP2025.10.31.685876v1-supplement-1.pdf]

bioRxiv preprint doi: https://doi.org/10.1101/2025.10.31.685876; this version posted November 3, 2025. The copyright holder for this preprint (which was not certified by peer review) is the author/funder, who has granted bioRxiv a license to display the preprint in perpetuity. It is made available under aCC-BY 4.0 International license.

Supplementary Information

Contents

Page

1 Sequence Processing **11**
1.1 Source Data . . . 11
1.2 Lineage and Germline Calling . . . 11

2 Quantitative Analysis **12**
2.1 Sampling Fraction and Interpretation of UMIs . . . 12
2.2 Analysis of Antibody-Secreting Cells . . . 13
2.3 Clonal Burst Analysis . . . 13
2.4 T Cell Spatial Analysis . . . 14
2.5 Error Estimation . . . 15

3 Phylogenetic Analysis **15**
3.1 Tree Construction and Node Inference . . . 15
3.2 Inferring Migration Rates . . . 16
3.3 Synthetic Data Generation . . . 16
3.3.1 Generating Migration Events . . . 16
3.3.2 Shuffling Migration Events . . . 17

List of Figures

S1 Statistics of clones across spatial barcodes. . . . 2
S2 The choice of threshold for lineage calling does not significantly impact our results. . . . 3
S3 Migrations still appear local after further data filtering. . . . 3
S4 Spatial distribution of T cell clones. . . . 4
S5 Analysis of ASC-dominated spatial barcodes. . . . 5
S6 Additional analysis of clonal bursts. . . . 5
S7 Lineage sizes follow a power law distribution with a slope that depends on their multifollicularity. . . . 6
S8 Distributions of inferred migration rates across lineages. . . . 7
S9 Criteria to identify lineages with recent clonal bursts. . . . 8
S10 Accounting for possible expansion of B cell lineages before GC entry. . . . 9
S11 Additional statistics of subtrees after migration events compared to null shuffled model. . . . 10
S12 UMI count and frequency of lineages in origin and destination follicles. . . 10

bioRxiv preprint doi: https://doi.org/10.1101/2025.10.31.685876; this version posted November 3, 2025. The copyright holder for this preprint (which was not certified by peer review) is the author/funder, who has granted bioRxiv a license to display the preprint in perpetuity. It is made available under aCC-BY 4.0 International license.

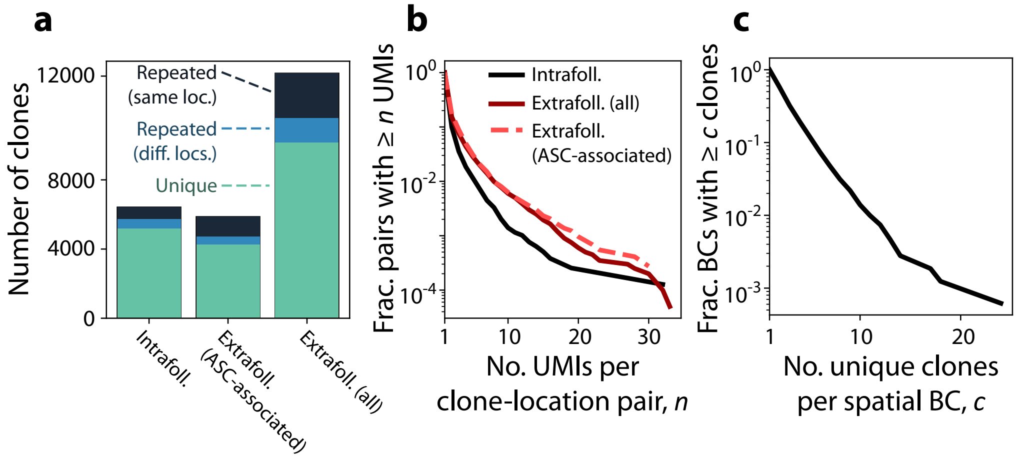

Figure S1: Statistics of clones across spatial barcodes. **(a)** Bar chart of the number of clones (distinct heavy chain VDJ sequences) observed within follicles and in the extrafollicular space, including the subset of spatial barcodes with ASC-associated RNA expression (SI 2.2). Charts are split into unique clones with only one observed UMI, clones with multiple UMIs all associated with different spatial barcodes, and clones with multiple UMIs in the same spatial barcode. **(b)** Survival function of the size of each clone, with the same delineation into groups as in (a). **(c)** Survival function of the number of unique clones associated with the same intrafollicular spatial barcode.

bioRxiv preprint doi: https://doi.org/10.1101/2025.10.31.685876; this version posted November 3, 2025. The copyright holder for this preprint (which was not certified by peer review) is the author/funder, who has granted bioRxiv a license to display the preprint in perpetuity. It is made available under aCC-BY 4.0 International license.

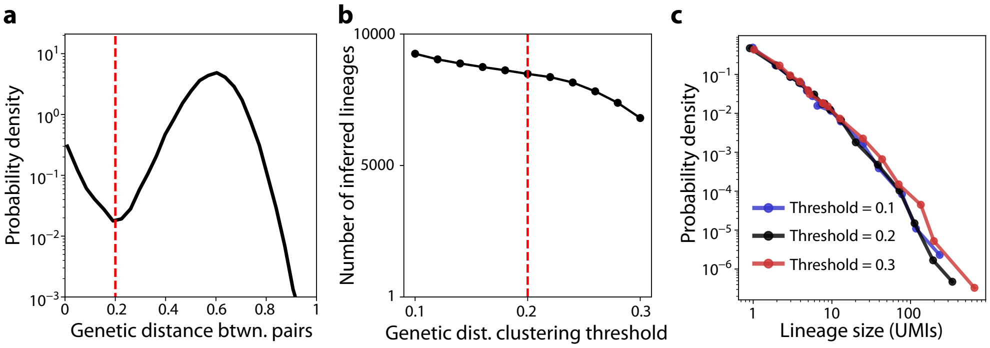

Figure S2: The choice of threshold for lineage calling does not significantly impact our results. **(a)** Distribution of CDR3 Hamming distances between all pairs of unique IGH reads in the same CDR3 group (SI 1.2). Dashed red line shows the threshold below which two sequences are called as part of the same lineage, after additional verification based on VJ sequence similarity (SI 1.2). **(b)** The number of inferred IGH lineages as a function of the lineage calling threshold in (a). Red dashed line shows the true threshold. **(c)** Distribution of IGH lineage sizes for different choices of lineage calling threshold. Note that all plots include both extrafollicular and intrafollicular reads.

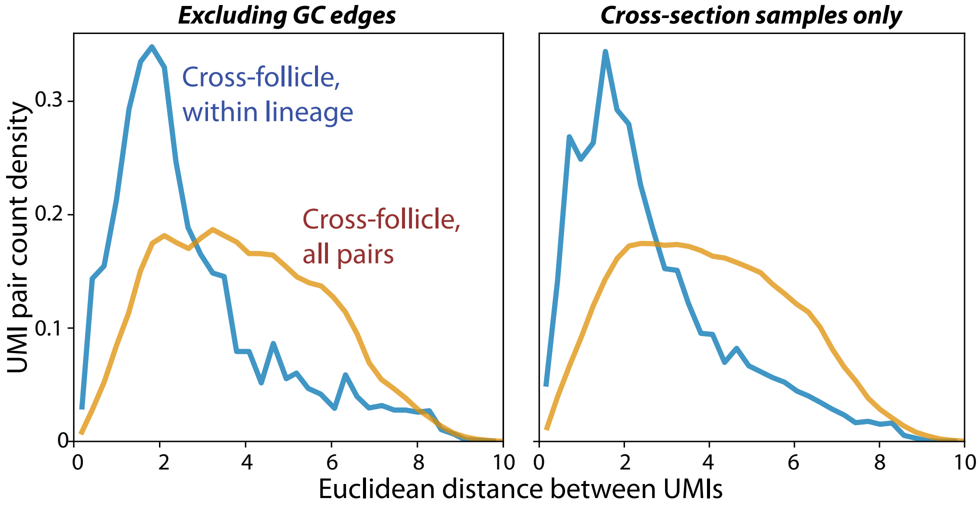

Figure S3: Migrations still appear local after further data filtering. Probability density of Euclidean distance (projected to the XY-plane) between UMIs corresponding to intrafollicular B cells, within lineages (blue) and across all pairs (orange). Left: Intrafollicular reads with spatial barcodes adjacent to extrafollicular spatial barcodes were omitted, to account for the possibility of ambiguous intrafollicular/extrafollicular labeling. Right: Only pairs of UMIs in different tissue sections were considered, accounting for the possibility of diffusion or contamination within a tissue section. In both plots, distance is normalized to the typical intrafollicular distance as in Fig. 1F.

bioRxiv preprint doi: https://doi.org/10.1101/2025.10.31.685876; this version posted November 3, 2025. The copyright holder for this preprint (which was not certified by peer review) is the author/funder, who has granted bioRxiv a license to display the preprint in perpetuity. It is made available under aCC-BY 4.0 International license.

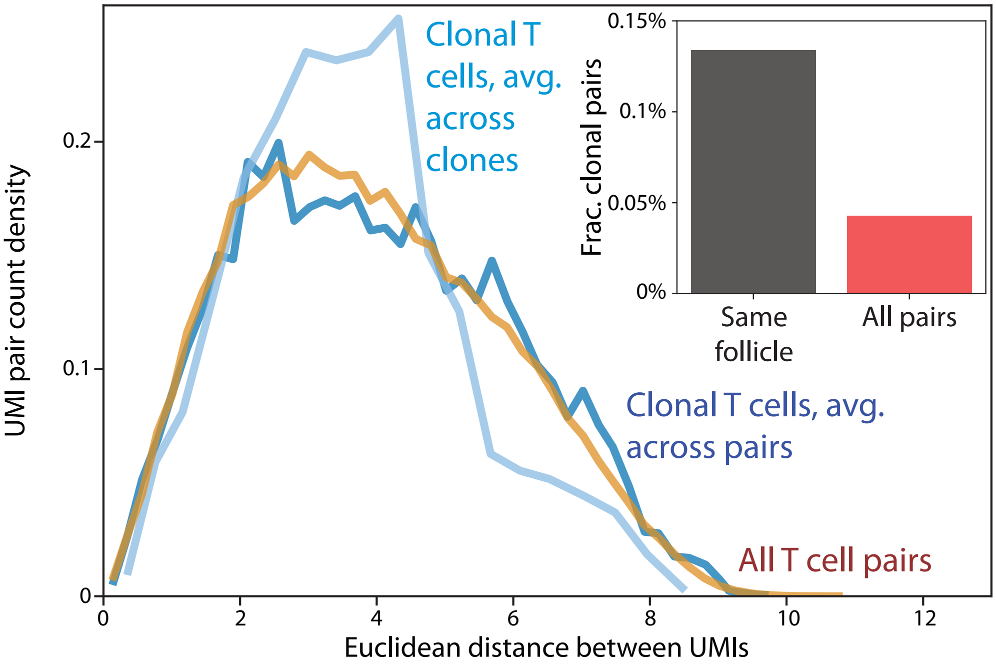

Figure S4: Spatial distribution of T cell clones. Probability density of Euclidean distance (projected to the XY-plane) between UMIs corresponding to intrafollicular TCRB reads in different follicles. Orange curve shows average over all pairs in different follicles, while dark blue curve shows average over clonal pairs (as in Fig. 1F). Light blue curve shows the distribution for clonal pairs, but where the average is first performed within each clone before averaging across clones, which has the effect of giving large clones the same weight as small clones. Distance is normalized to the typical intrafollicular distance as in Fig. 1F. Inset: probability that a random pair of TCRB UMIs correspond to a clonal sequence, conditional on being in the same follicle (black) or not (red). Note that the majority of the difference between the bars comes from clonal UMIs in the same spatial barcode.

bioRxiv preprint doi: https://doi.org/10.1101/2025.10.31.685876; this version posted November 3, 2025. The copyright holder for this preprint (which was not certified by peer review) is the author/funder, who has granted bioRxiv a license to display the preprint in perpetuity. It is made available under aCC-BY 4.0 International license.

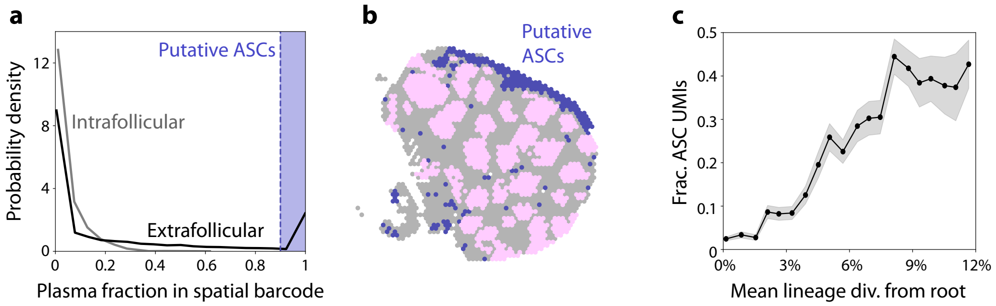

Figure S5: Analysis of ASC-dominated spatial barcodes. **(a)** Distribution of plasma fraction (SI 2.2) for intrafollicular and extrafollicular spatial barcodes. Blue dashed line shows the cutoff where we defined spatial barcodes as ASC-dominated. **(b)** Locations of ASC-dominated spatial barcodes in one tissue section (shown in blue). **(c)** Lineages were binned by mean divergence from the inferred root sequence among their intrafollicular UMIs – a proxy for their age. Plot shows the proportion of UMIs in each lineage located in ASC-associated extrafollicular spatial barcodes (SI 2.2), as a function of this binned intrafollicular age. Extrafollicular reads in locations not strongly associated with ASC RNA expression were excluded.

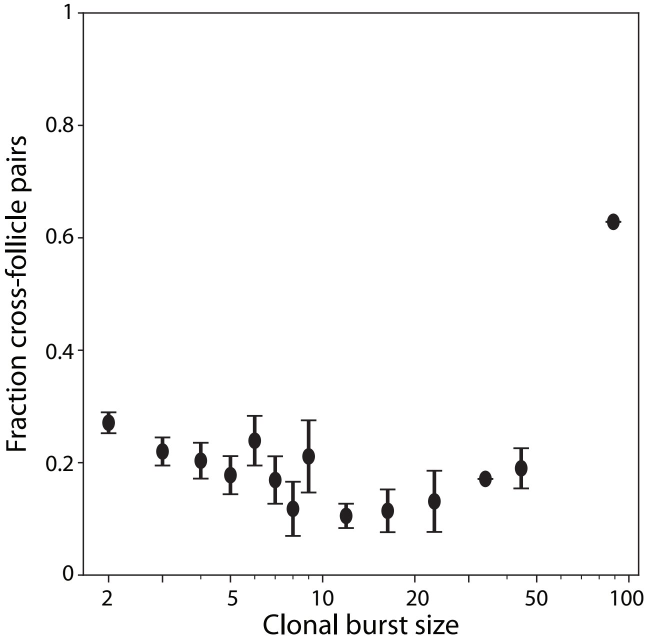

Figure S6: Additional analysis of clonal bursts. Probability that any pair of UMIs in a clonal burst are in different follicles, as a function of the binned clonal burst size. Error bars show standard deviation across different clonal bursts. Clonal bursts are defined as UMIs with the same V sequence with at least 1% divergence from their inferred germline ancestor that appears at least twice within GCs.

bioRxiv preprint doi: https://doi.org/10.1101/2025.10.31.685876; this version posted November 3, 2025. The copyright holder for this preprint (which was not certified by peer review) is the author/funder, who has granted bioRxiv a license to display the preprint in perpetuity. It is made available under aCC-BY 4.0 International license.

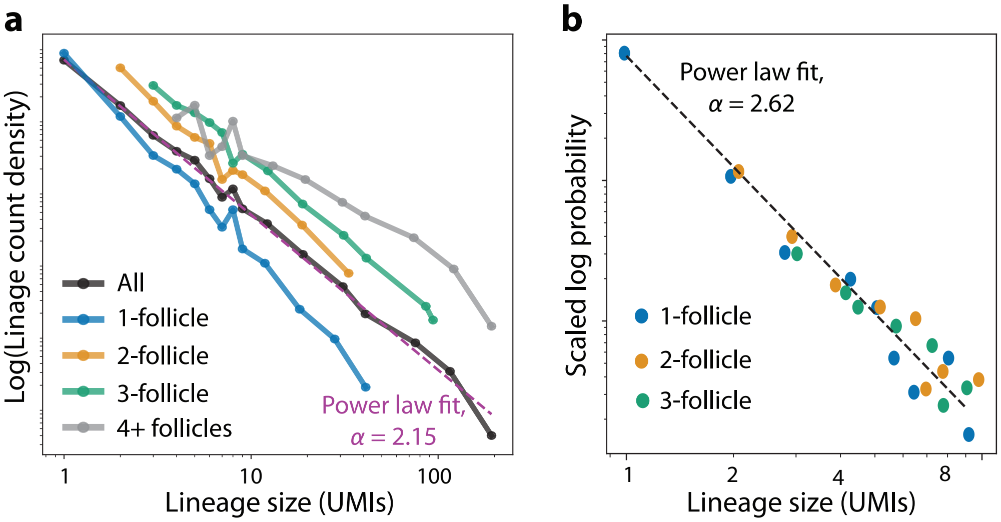

Figure S7: Lineage sizes follow a power law distribution with a slope that depends on their multifollicularity. (a) Distribution of B cell lineage sizes (number of intrafollicular UMIs) conditional on the number of follicles they are found in. Purple dashed line shows a power law fit to the entire distribution, $y \sim x^{-\alpha}$. **(b)** The 1-, 2-, and 3-follicle distributions from (a) translated to pass through the same point, truncated at 9 UMIs. Dashed line shows a single power law fit to all three distributions simultaneously, resulting in a steeper slope than the full distribution in (a).

bioRxiv preprint doi: https://doi.org/10.1101/2025.10.31.685876; this version posted November 3, 2025. The copyright holder for this preprint (which was not certified by peer review) is the author/funder, who has granted bioRxiv a license to display the preprint in perpetuity. It is made available under aCC-BY 4.0 International license.

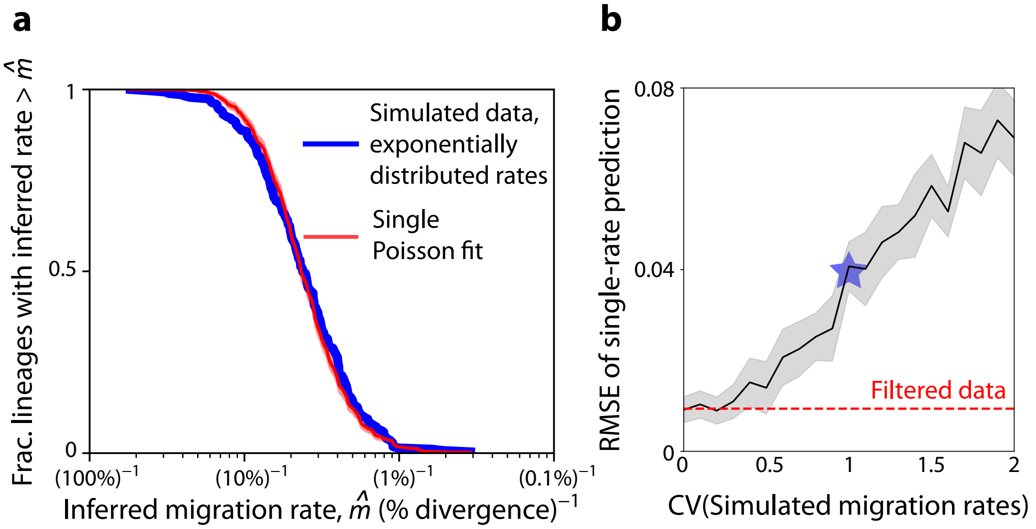

Figure S8: Distributions of inferred migration rates across lineages. **(a)** Survival function for a simulated dataset where migration rates are lineage-specific, drawn from an exponential distribution with mean equal to the inferred rate from Fig. 3C (SI 3.3.1). Red curve shows the best-fit survival function for a model with a single Poisson rate across all lineages, which is underdispersed relative to the simulated data. **(b)** Root mean squared error of the best-fit single-rate survival function for a range of simulated data where migration rates are lineage-specific, drawn from a gamma distribution with the same mean as before, and a range of standard deviations. Shaded region shows standard deviation of the error across 10 simulations. Red line shows the RMSE of the real data relative to the single-rate fit, after filtering out putative clonal bursts as in the inset of Fig. 3C. Blue star shows a coefficient of variation of 1, corresponding to the blue curve in (a).

bioRxiv preprint doi: https://doi.org/10.1101/2025.10.31.685876; this version posted November 3, 2025. The copyright holder for this preprint (which was not certified by peer review) is the author/funder, who has granted bioRxiv a license to display the preprint in perpetuity. It is made available under aCC-BY 4.0 International license.

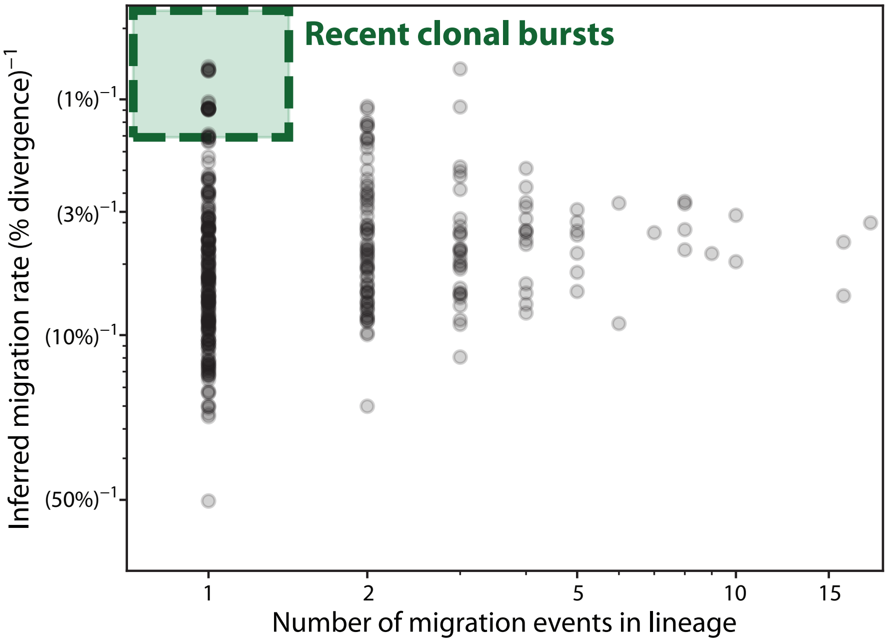

Figure S9: Criteria to identify lineages with recent clonal bursts. Scatter plot of the inferred migration rate of each lineage and the actual number of identified migration events. Green box shows the lineages identified as having had recent clonal bursts: lineages with only one migration event that were nonetheless in the top 10% of inferred migration rates.

bioRxiv preprint doi: https://doi.org/10.1101/2025.10.31.685876; this version posted November 3, 2025. The copyright holder for this preprint (which was not certified by peer review) is the author/funder, who has granted bioRxiv a license to display the preprint in perpetuity. It is made available under aCC-BY 4.0 International license.

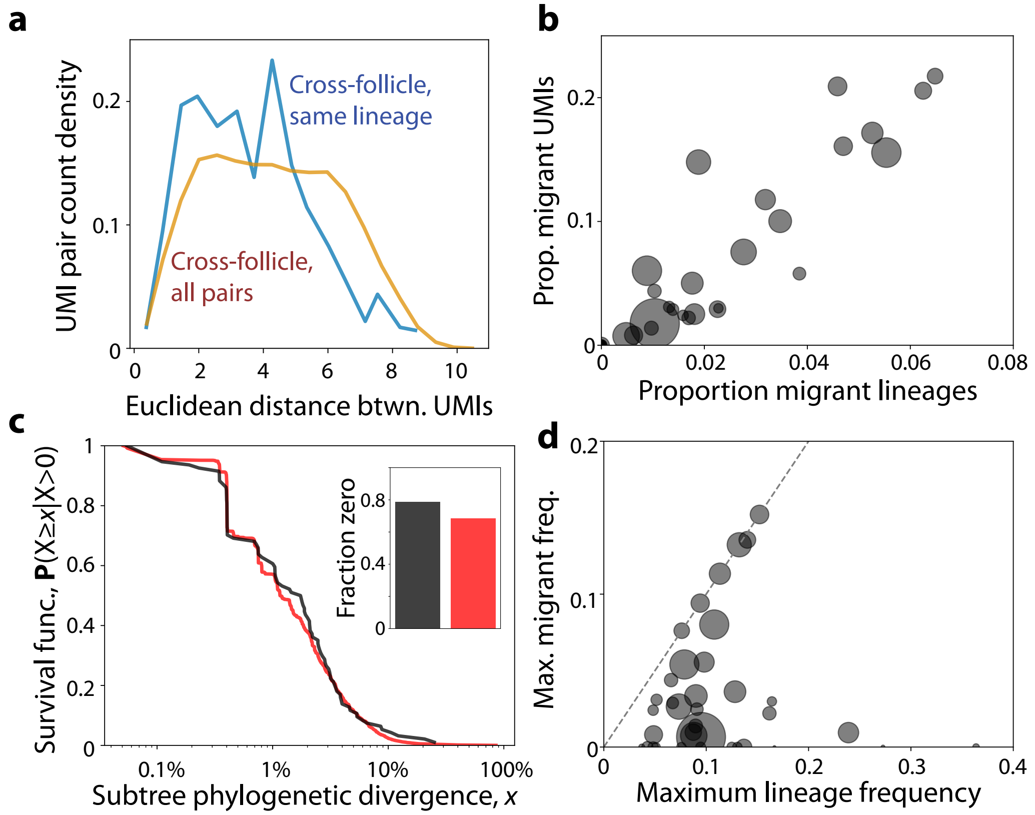

Figure S10: Accounting for possible expansion of B cell lineages before GC entry. (a) Distribution of Euclidean distance between UMIs which are both below 1% divergence from their inferred root sequence, comparing members of the same lineage to all pairs of UMIs. Distance was measured and normalized to the typical within-GC length scale (dashed line) as in Fig. 1F. **(b)** Phylogenetic divergence of subtrees after migration events, as in Fig. 5A, ignoring migration events that could have occurred before the 1% divergence cutoff (SI 3.3.2). **(c-d)** Proportion of migrant UMIs and their frequencies as in Fig. 5B-C, where lineages were only counted as migrants if they migrated after reaching 1% divergence from their root (filtering out possible expansion before GC entry).

bioRxiv preprint doi: https://doi.org/10.1101/2025.10.31.685876; this version posted November 3, 2025. The copyright holder for this preprint (which was not certified by peer review) is the author/funder, who has granted bioRxiv a license to display the preprint in perpetuity. It is made available under aCC-BY 4.0 International license.

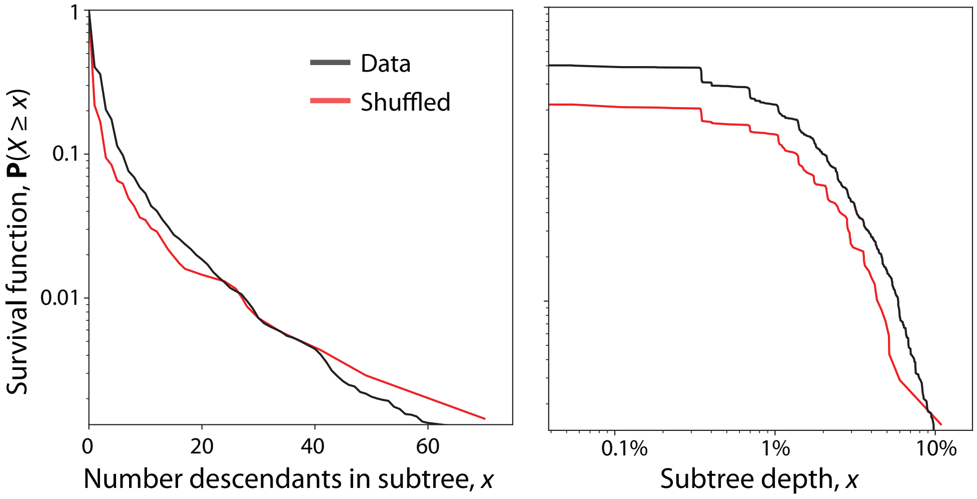

Figure S11: Additional statistics of subtrees after migration events compared to null shuffled model. Statistics of subtrees after migration events compared to a shuffled model, as described in Fig. 5A, but using statistics other than phylogenetic divergence. Left: survival function of the total number of unique members of the subtree, after the first. Right: survival function of the total depth of the subtree. Red curves show shuffled data as in Fig. 5A.

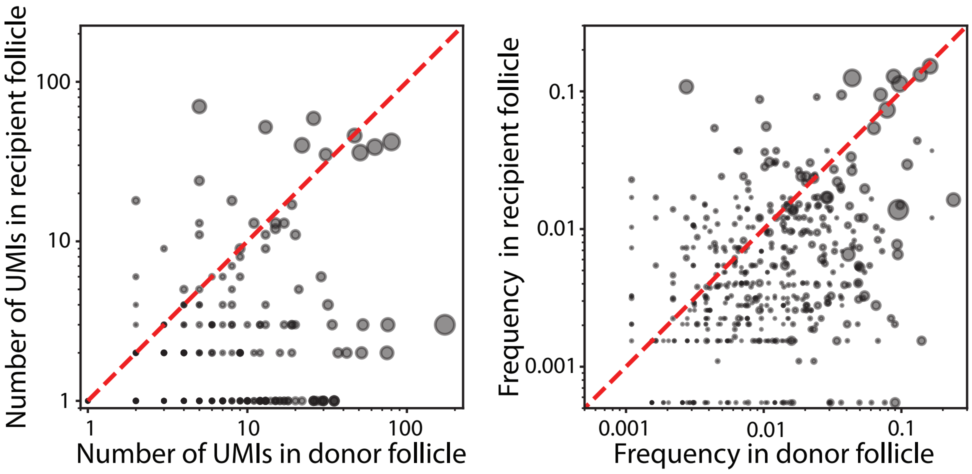

Figure S12: UMI count and frequency of lineages in origin and destination follicles. Scatterplot of the count (left) and frequency (right) of each migratory lineage with at least two distinct sequences, in its origin follicle (the inferred follicle of its germline ancestor) and the non-origin follicle where its count/frequency was greatest. Point size is proportional to total lineage size. Red dashed line shows $y = x$.

bioRxiv preprint doi: https://doi.org/10.1101/2025.10.31.685876; this version posted November 3, 2025. The copyright holder for this preprint (which was not certified by peer review) is the author/funder, who has granted bioRxiv a license to display the preprint in perpetuity. It is made available under aCC-BY 4.0 International license.

1 Sequence Processing

1.1 Source Data

Data was sourced from the long-read Spatial VDJ tonsil data in Ref. (16), accessible via Zenodo (21). Specifically, the following files from the `SpatialVDJ_forZenodo/data/tonsil/1_LR-SpatialVDJ/` directory were used:

1. `read_lists/UMI_collapsed_on_tissue_read_list.csv`
2. `metadata/tonsil_LR_spatialbc_metadata.csv`
3. `clone_list/tonsil_all_ontissue_clone_list.csv`

File 1 was the main input to our data analysis pipeline to analyze heavy chain B cell sequences. File 2 was used for associations between spatial barcodes, spatial positions and local RNA expression data used to infer plasmablast gene expression. File 3 was used only to label T cell clones from File 1 for T cell spatial distribution analysis.

1.2 Lineage and Germline Calling

The data analysis pipeline to filter sequences, cluster them into lineages and annotate them with their inferred germline ancestor was largely the same as described in SI Section C.1 of Ref. (4). A Snakemake version of the pipeline is available on Github (`https://github.com/icvijovic/tonsil-spatial`). In brief, consensus reads were read from File 1, discarding reads with ambiguous bases. Then, `IgBLAST` was used to annotate sequences, followed by a filtering step to retain only high-quality VDJ transcripts. Finally, sequences were clustered into lineages and assigned a putative germline sequence, in a manner detailed below.

In order to cluster sequences into putative lineages, sequences were grouped into "CDR3 groups" with the same CDR3 length and V gene family. Single linkage clustering was performed within CDR3 groups, with a threshold of 0.2 for the CDR3 nucleotide Hamming distance. These putative lineages were further clustered to obtain the final lineage IDs, imposing the additional requirement that members of the same lineage have a fractional Levenshtein distance of less than 0.2 between their templated regions. This threshold of 0.2 was chosen based on the CDR3 pairwise Hamming distance distribution among IGH sequences in the same CDR3 group (Fig. S2A). This distribution has two peaks which roughly separate the pairs into related and unrelated sequences, with the threshold chosen as the dividing line. Note that the threshold of 0.2 is different from Ref. (4), which used a threshold of 0.15. However, the number of inferred lineages and the size distribution of lineages are not extremely sensitive to the chosen threshold, suggesting our results are unlikely to depend strongly on ambiguous lineage calls (Fig. S2B-C).

As in Ref. (4), germline annotations were performed using `grmlin` (23), which generates a proposed database of V gene germline sequences based on the data. Sequences were

bioRxiv preprint doi: https://doi.org/10.1101/2025.10.31.685876; this version posted November 3, 2025. The copyright holder for this preprint (which was not certified by peer review) is the author/funder, who has granted bioRxiv a license to display the preprint in perpetuity. It is made available under aCC-BY 4.0 International license.

then re-aligned to the new V gene database using BLAST. Again following the procedure of Ref. (4), the output database was polished by adding additional putative germline sequences from the complete IMGT database (24), which were undetected by grmlin. Sequences were only added to the inferred database if they were sufficiently distant from existing germline sequences and supported by multiple lineages. After refinement of this germline database, sequences were once again aligned to the polished germline. The output of this pipeline is the tonsil_vdjs_annotated.tsv file used for the majority of our downstream analysis.

As a last data cleanup step, we checked to ensure that all lineages had a single consensus germline sequence. All lineages that contained members with multiple V gene calls were split into new lineages with a single V call, resulting in 151 new putative lineages. Two additional lineages had members whose putative V sequences had different lengths and could not be resolved by padding the start of the sequence with unknown nucleotides; these lineages were also split. Seven reads with V gene mutation calls that included indels were removed from the dataset. Finally, we accounted for the possibility that BLAST had truncated mutations near the ends of the V gene in some reads by updating them according to a single consensus sequence, based on the most common sequence inferred among the members of each lineage. The final dataset has 8634 lineages, 3215 of which have members in the intrafollicular space – the focus of our analysis.

2 Quantitative Analysis

2.1 Sampling Fraction and Interpretation of UMIs

Unique molecular identifiers (UMIs) are individual barcodes associated with individual mRNA reads. Comparing the number of UMIs associated with different mRNA sequences provides a way to estimate their relative abundances in the dataset, before biases are introduced by RNA amplification. However, many of our stochastic models interpret statistics on the level of individual cells and not individual mRNA molecules, motivating us to estimate the correspondence between these quantities.

Most unique VDJ sequences (clones) in the dataset are observed only once (Fig. S1A). An even smaller fraction (about 10% of intrafollicular clones) are ever observed multiple times associated with the same spatial barcode (Fig. S1A-B). We expect many of the sequences repeatedly sampled in the same region nonetheless correspond to multiple clonally related cells, because spatial barcodes span a wide enough region to typically include several cells (Fig. S1C). Indeed, 42% of these sequences were sampled across multiple spatial barcodes (data not shown), consistent with a clone expanded to many cells, rather than a single cell sampled multiple times. We can place a rough upper bound on the probability of sampling a second VDJ mRNA molecule in an already-sampled cell as 4% – the fraction of sequences observed exactly twice, both times with the same associated spatial barcode. Even a substantial fraction of this 4% likely result from independently sampling distinct cells. Thus, we generally interpret intrafollicular UMIs in this data as individual cells, assuming that "double-counting" rates are small enough to not significantly bias our data.

bioRxiv preprint doi: https://doi.org/10.1101/2025.10.31.685876; this version posted November 3, 2025. The copyright holder for this preprint (which was not certified by peer review) is the author/funder, who has granted bioRxiv a license to display the preprint in perpetuity. It is made available under aCC-BY 4.0 International license.

A corollary of small double-counting rates per cell is that the probability of detecting an individual cell to begin with is likely much less than unity. If this is the case, the dataset will only reflect a fraction $\eta \ll 1$ of the cells present in the initial tonsil slices. We can estimate this sampling fraction by comparing the number of VDJ mRNAs typically sampled per cell to other methods. In putative ASCs, which are typically characterized by high levels of VDJ expression, 10X VDJ sequencing typically detects 100-1000 UMIs per cell (4). While we lack individual cell resolution in the Spatial VDJ data, the number of UMIs observed associated with the same clone in the same spatial barcode places an upper bound on the per-cell UMI number, as discussed above. While this quantity is elevated for putative ASCs in the Spatial VDJ data (SI 2.2) relative to intrafollicular B cells, it is still rare to observe more than ∼10 UMIs per VDJ per spatial barcode (Fig. S1B). Thus, we expect that the sampling fraction is no more than $\eta \sim 1\%$. Because B cells typically express the heavy chain at levels 2-3 orders of magnitude lower than ASCs, this also implies that many lineages and clones are left undetected, and the sizes of many detected clones are much larger than their sampled size would suggest. While most of our analyses will be roughly independent of this sampling fraction, it is important to remember that the size of lineages in the dataset is likely much less than their true size in the tonsil.

2.2 Analysis of Antibody-Secreting Cells

While most of our analysis focuses on the intrafollicular B cells presumed to be undergoing affinity maturation, Fig. 2B, Fig. S1 and Fig. S5 analyze cells inferred to be ASCs that have exited affinity maturation. To identify these cells, spatial barcode metadata from File 2 was used to calculate the "plasma fraction" for each barcode:

$$\text{Plasma frac} \equiv \frac{p}{b+p}, \tag{S1}$$

where p is the *stereoscope* cell type label associated with plasmablasts inferred in Ref. (16), and b is the corresponding quantity for B cells. UMIs in extrafollicular spatial barcodes with a plasma fraction of at least 0.9 were assumed to be associated with ASCs. Note that Fig. S1B suggests there is little to no systematic difference in the number of UMIs associated with these putative ASCs and all extrafollicular UMIs, likely indicating that there are many ASCs missed by this conservative local gene expression threshold.

2.3 Clonal Burst Analysis

Our analysis in Fig. 2D and Fig. S6 focuses on clonal bursts, which we define as VDJ sequences which are more than 1% diverged from their germline sequence, and appear associated with more than one UMI. Following our analysis in SI 2.1, we interpret these UMIs as distinct cells. Because these clones are genetically identical, inferring the exact phylogeny within a clonal burst is not possible. Instead, we group clonal bursts by their size to determine whether there are consistent properties among clonal bursts of similar size.

Accounting for the detection threshold discussed in SI 2.1, a clonal burst of M observed cells actually represents $\sim M/\eta$ cells in the tonsil, such that the typical pair of cells in

bioRxiv preprint doi: https://doi.org/10.1101/2025.10.31.685876; this version posted November 3, 2025. The copyright holder for this preprint (which was not certified by peer review) is the author/funder, who has granted bioRxiv a license to display the preprint in perpetuity. It is made available under aCC-BY 4.0 International license.

the burst is separated by $\sim \log_2(M/\eta)$ cell divisions. If a migration event has a constant, independent chance of occurring each cell division, this means that pairs of UMIs in larger clonal bursts should have a higher probability of being in separate follicles, scaling with $\log_2(M)$. However, this prediction is not consistent with the data, which instead exhibits a roughly constant pairwise migration probability over a wide range of M (Fig. S6). This result is consistent with a model where each cell produced during the clonal burst has an independent probability of migrating, irrespective of the total number of cells produced. One biological interpretation of this model is that clonal bursts occur in the dark zone during a single light zone/dark zone cycle, and cells only have a chance to migrate after the clonal burst is complete and cells re-renter the light zone. In this case, migration would not have a chance of occurring each cell division, but rather during each LZ/DZ cycle. This motivates the geometric fit in Fig. 2D, representing a model where each cell has an independent $\sim 7\%$ chance of migrating in a clonal burst.

If clonal bursts occur in a single LZ/DZ cycle, we can roughly compare the migration rate during a clonal burst to the long-term migration rate of one every 50-70 cell divisions estimated elsewhere in our paper. Typical cells divide about 2 times per cycle (34), in which case our earlier estimate of one migration every 50-70 cell divisions corresponds to a 3-4% migration chance per cell per cycle. This is somewhat lower than the 7% chance predicted during clonal bursts, but not drastically different, and within the uncertainty of these rough approximations (such as assuming a constant number of divisions per cycle for non-bursting cells). Thus, migration during clonal bursts occurs at similar rates to typical cells. However, because large clonal bursts produce many cells which each have a chance to migrate, the chance that at least one of these cells migrates can be substantial.

2.4 T Cell Spatial Analysis

Our analysis of the B cell migration process raises the question of how it compares to T cell migration. The spatial distribution of cross-follicle T cell clones in the tonsil appears much more well-mixed than B cells (Fig. 1F), indicating they migrate at higher rates or across longer distances. However, the presence of spatially widespread clones could arise from T cell migration or proliferation of T cells before activation and GC entry (31, 37) – processes which are difficult to decouple in this data, because T cells lack hypermutations which can be used to estimate the time they have spent in the affinity maturation process. Consistent with the possibility of proliferation before GC entry, smaller T cell clones do not appear as well-mixed across the tonsil as larger clones (Fig. S4), suggesting that some of the largest T cell clones may owe their wide spatial distribution to having been seeded in multiple germinal centers. Despite the difficulties of estimating the T cell migration rate from a single snapshot, we can conclude that it is likely less than the T cell division rate, based on the fact that T cells in the same follicle are significantly more likely to be related than T cells in distinct follicles (Fig. S4, inset). This is consistent with results in mice, where the T cell migration rate between GCs has been measured to be $\sim$1/60 hr (31), or roughly $\sim$1/6 cell divisions (32, 33). If the T cell migration rate is comparable in the human tonsil, the ratio of migration to division rates would be about an order of magnitude smaller in B cells than in T cells. Regardless of the precise migration rate, we can conclude that a combination

bioRxiv preprint doi: https://doi.org/10.1101/2025.10.31.685876; this version posted November 3, 2025. The copyright holder for this preprint (which was not certified by peer review) is the author/funder, who has granted bioRxiv a license to display the preprint in perpetuity. It is made available under aCC-BY 4.0 International license.

of migration and independent GC entry results in a distribution of T cell clones across the tonsil which is much more well-mixed than B cells.

2.5 Error Estimation

In order to generalize the conclusions we draw from Ref. (16) to statistically similar datasets, it is sometimes useful to estimate the error in our calculated probabilities and frequencies. In most figures with error bars, we show counting error Δy (square root of the number of counts of lineages, UMIs, etc.), which indicates how many data points contributed to a given estimate. On a linear scale, we plot error bars on the interval $(y - \Delta y,\ y + \Delta y)$; on a log scale, we propagate it as $(ye^{-\Delta y/y},\ ye^{\Delta y/y})$.

3 Phylogenetic Analysis

3.1 Tree Construction and Node Inference

We used FastTree version 2.1.11's nucleotide alignment (25, 26) to construct phylogenetic trees for each inferred lineage with at least two distinct intrafollicular VDJ sequences. The tree was rooted at the inferred germline sequence (SI 1.2), which was added to the pool of intrafollicular sequences in the lineage if it was not present already. This results in a tree for each lineage with branch lengths roughly corresponding to sequence divergence. Note that extrafollicular sequences were excluded from this analysis.

To study migrations along the tree, we assigned inferred follicular locations to internal (unobserved) nodes. Specifically, each terminal node was labeled with its follicular location in the dataset. If a node was associated with a sequence found in multiple follicles, we chose the follicle it most frequently appeared in, making a random choice if there was a tie. Then, a version of Fitch maximum parsimony (28) was used to infer internal node states. Starting at the bottom of the tree, each undetermined node was assigned a set of possible locations equal to the intersection of its children's locations, or their union if no intersection existed. Then, from the top of the tree working down, we chose one of the possible locations for each node, matching the parent's chosen location if possible.

Note that the top-down step often requires choices between possible locations which cannot be resolved by maximum parsimony alone – most obviously, inferring the follicular location of a node with two descendants in different follicles. To remedy this issue, when a choice between possibilities had to be made, we assumed that a node was located in the follicle that the majority (in terms of UMIs) of its observed descendants were in. If there was still a tie in UMI number, we chose the follicle that its descendants had the highest frequency in (i.e., the follicle with the smaller number of total UMIs). Effectively, these assumptions conservatively estimate the impact of migrations: when migration locations are not constrained by the topology of the tree, we assume the source follicle is the one with higher UMI count or frequency.

bioRxiv preprint doi: https://doi.org/10.1101/2025.10.31.685876; this version posted November 3, 2025. The copyright holder for this preprint (which was not certified by peer review) is the author/funder, who has granted bioRxiv a license to display the preprint in perpetuity. It is made available under aCC-BY 4.0 International license.

3.2 Inferring Migration Rates

In Fig. 3, we inferred migration rates based on the trees constructed following the procedure in SI 3.1. Specifically, the rate was estimated as the number of migration events n_{mig} divided by the total phylogenetic divergence of the tree T (i.e., the sum of all branch lengths). This procedure resulted in the tree-specific migration rates used in Fig. 3C. Note that many of these individual rates are highly imprecise, because they are inferred from a small number of migration events. This uncertainty can be quantified as a 90% confidence interval assuming that the migrations arise from a Poisson process. Specifically, the migration rate bounds $\hat{m}_{\pm}$ satisfy the equations

$$\sum_{n=0}^{n_{\mathrm{mig}}} \mathcal{P}_{\mathrm{Poiss}}(n, T\hat{m}_{+}) = 0.05, \tag{S2}$$

$$\sum_{n=n_{\mathrm{mig}}}^{\infty} \mathcal{P}_{\mathrm{Poiss}}(n, T\hat{m}_{-}) = 0.05, \tag{S3}$$

where $\mathcal{P}_{\mathrm{Poiss}}(n, \lambda)$ is the Poisson PMF with rate λ evaluated at n. In practice, we usually investigate the distribution of $\hat{m}$ across lineages through its survival function, whose shape reflects the noise in the dataset in a more controlled way. The same general approach can be used to calculate other conditional migration rates, depending on the trees or portions of trees included in the denominator. To produce Fig. S10A, we estimated the age of each migration event as the average divergence from germline on the branch where the migration occurred, and binned migration events by their age. To convert from migration counts to migration rates, we summed together the branch lengths of *all* branches in that age range (across all lineages), regardless of whether a migration event occurred on it. This gives the phylogenetic divergence that serves as the denominator for migration rates, correcting for biases in how many branches are observed within a given divergence range.

We also generated an alternative survival function of migration rates, omitting lineages which might correspond to clonal bursts (Fig. 3C, inset). Specifically, we removed lineages from the dataset whose migration rates were in the top 10% of inferred migration rates, but were only based on one migration event (Fig. S9). This indicates that their sole migration happened in a very short phylogenetic divergence – suggesting they were due to recent clonal bursts. Because these estimates were based on only one migration event, their 90% confidence intervals are also very wide.

3.3 Synthetic Data Generation

3.3.1 *Generating Migration Events*

A related method used in our analysis was to generate synthetic trees with the same phylogenetic structure, but a different arrangement of migration events. To generate the red curve in Fig. 3C, we took the same trees as in the actual dataset, but sampled the number of migration events that occurred along them according to a Poisson distribution with a characteristic rate $\hat{m}$ per genetic divergence. This migration count was then used as input

bioRxiv preprint doi: https://doi.org/10.1101/2025.10.31.685876; this version posted November 3, 2025. The copyright holder for this preprint (which was not certified by peer review) is the author/funder, who has granted bioRxiv a license to display the preprint in perpetuity. It is made available under aCC-BY 4.0 International license.

to re-infer lineage-specific migration rates as in SI 3.2. The survival function of these rates (omitting those with no migration events) was compared to the true distribution via its L2 error,

$$\text{Err}(\hat{m}) \equiv \frac{1}{N_{\text{trials}} N_{\text{pts}}} \sum_{i=1}^{N_{\text{trials}}} \sum_{j=1}^{N_{\text{pts}}} \left[s_i - \hat{s}_{ij}(\hat{m})\right]^2 , \tag{S4}$$

where s_i and $\hat{s}_i$ are the values of the survival function at a set of migration rates indexed by i for the real and synthetic data, respectively. Synthetic data was generated $N_{\text{trials}} = 100$ times for each value of $\hat{m}$ tested, and the optimal $\hat{m}^*$ was chosen based on the vertex of a quadratic fit near the tested $\hat{m}$ that minimized the error. The optimal $\hat{m}^*$ was used for the red curve in Fig. 3C and as the model in Fig. 3D, under the assumption that a lineage is found in a number of follicles equal to the number of migration events it has experienced plus one.

In Fig. S8A, we generated migration events according to a more complex process, to test whether our analysis was sensitive to migration rate variability across lineages. Specifically, we sampled unique migration rates for each lineage from an exponential distribution with mean $\hat{m}^*$, then sampled migration events for each lineage according to that rate. The resulting distribution was clearly overdispersed relative to the true data, indicating that the true variability in migration rates is subexponential. We extended this analysis in Fig. S8B, drawing rates from a gamma distribution with mean $\hat{m}^*$ and a range of standard deviations, and quantifying how well the resulting survival function matched the best-fit single-rate curve through root mean square error.

3.3.2 *Shuffling Migration Events*

As an additional null model for migration along a phylogenetic tree, we considered trees with identical topology to the true trees, but with uniformly shuffled migration events. After labeling the internal nodes of each tree as described in SI 3.1, we counted the number n of migration events it contained (i.e., branches where the location of the parent and child were different), neglecting clonal bursts. We then chose n random positions on the tree for migrations to occur, mapping each branch on the tree to a position on the number line from 0 to 1. This corresponds to a model where migrations have a constant probability over evolutionary time, but are otherwise independent of the structure of the tree. Then, we re-assigned follicular locations on the random tree corresponding to the shuffled events. By performing this procedure 100 times for each tree topology, we were able to produce a “null distribution” of randomly shuffled migration events along the tree.

This procedure was used to generate the red curve in Fig. 5A and Fig. S11. Subtrees were defined as the portion of a tree following a branch with a migration event, but not containing any migration events itself – i.e., regions inferred to be entirely local to one follicle (Fig. 5A, schematic). An extension of this procedure was used for Fig. S10C, ignoring branches which began below 1% divergence (both for the purposes of calling migration events, and shuffling them).
